# Systematic review and meta-analysis of vitamin D deficiency in different pregnancy on preterm birth: Deficiency in middle pregnancy might be at risk

**DOI:** 10.1097/MD.0000000000026303

**Published:** 2021-06-18

**Authors:** Rui-Han Lian, Ping-An Qi, Tao Yuan, Pei-Jing Yan, Wen-Wen Qiu, Ying Wei, Ya-Guang Hu, Ke-Hu Yang, Bin Yi

**Affiliations:** a Gansu Provincial Maternity and Child-Care Hospital; b Gansu University of Chinese Medicine; c The First Hospital of Lanzhou University; d Institute of Clinical Research and Evidence Based Medicine, Gansu Provincial Hospital; e Evidence-Based Medicine Center, School of Basic Medical Sciences, Lanzhou University; f Key Laboratory of Evidence Based Medicine and Knowledge Translation of Gansu Province, Lanzhou, China.

**Keywords:** different periods of gestation, meta-analysis, preterm birth, vitamin D deficiency

## Abstract

**Background:**

Current studies suggest that vitamin D deficiency during pregnancy can produce a certain effect for preterm birth (PTB), but there is no research showing whether vitamin D deficiency has a consistent effect in different pregnancies; thus, we conducted a systematic review and meta-analysis of 24 observational studies, grouping them according to the gestational age at the time of serum sampling, to investigate whether vitamin D deficiency in different periods of gestation has different effects on PTB and to provide an evidence-based basis for pregnant women to measure and supplement vitamin D.

**Methods:**

The databases PubMed-Medline, EMBASE, the Cochrane Library, Web of Science, EBSCO, CBM, and CNKI were searched until February 2020. Two researchers independently assessed the eligibility and quality of studies, and STATA 12.0 software was used for meta-analysis.

**Result:**

Seven cohort studies, 13 case–control studies, and 4 cross-sectional studies were included from 2500 articles by inclusion and exclusion criteria. After adjusting for age, race, and other confounding factors, meta-analysis results showed that vitamin D deficiency in the first trimester, the second trimester, and the third trimester did not increase the risk of PTB (odds ratio (OR) = 1.01, 95% confidence interval (CI) (0.88, 1.16), *P* = .867; OR = 1.12, 95%CI (0.92, 1.37), *P* = .249; OR = 1.05, 95%CI (0.87, 1.27), *P* = .602). However, there was moderate heterogeneity in the study of vitamin D deficiency in the second trimester, and subgroup analysis suggested that vitamin D deficiency in the second trimester may increase the risk of PTB (OR = 1.33, 95%CI (1.15, 1.54), *P* = .000). A sensitivity analysis of the second trimester showed that excluding any 1 study did not significantly change the results.

**Conclusions:**

Vitamin D deficiency in early and late pregnancy may not be associated with PTB, while vitamin D deficiency in middle pregnancy is likely to have an important effect on PTB. Vitamin D levels should be measured in the second trimester of pregnancy, and vitamin D supplements should be provided if necessary.

## 1 Introduction

The World Health Organization (WHO) defines preterm birth (PTB) as a baby born before 37 weeks of gestation, meaning fewer than 259 days from the first day of a woman's last menstrual period.^[1]^ PTB is the most common cause of neonatal death worldwide and the second most common cause of death among children under 5 years of age,^[2]^ and is 1 of the major health indicators in a country. According to the WHO, PTB rates in countries range from 5% to 18%.^[3]^ Because the fetus cannot realize its growth potential in utero,^[4]^ direct or hidden adverse consequences will be generated for future growth and development. The incidence of necrotizing enterocolitis, retinopathy, neonatal jaundice, hypoxic-ischemic encephalopathy, and other diseases in premature infants was significantly higher than in term infants. Up to 40% of premature survivors have bronchopulmonary dysplasia, while cerebral palsy, epilepsy, cognitive impairment, and other neurological diseases often occur in premature infants.^[5]^ Pregnant women with high-risk factors such as smoking, obesity, and gestational diseases are more likely to give birth prematurely, and even among healthy women, a certain percentage of babies may be born prematurely.^[4]^ The risk factors of PTB are complex, and the prevention of PTB is a global health problem.^[2,6]^

Vitamin D is a lipid-soluble metabolite that plays an important role in maintaining calcium and phosphorus homeostasis and promoting bone metabolism. In recent years, the role of vitamin D in extracellular health has been paid more and more attention. In terms of metabolism during pregnancy, there is a good deal of research and controversy regarding the effects of vitamin D levels during pregnancy on fetuses and neonates.^[7,8]^ Vitamin D deficiency during pregnancy is common around the world.^[8]^ It has been linked to an increased incidence of poor maternal and fetal outcomes, mainly preeclampsia, gestational diabetes, low birth weight, and PTB.^[9]^ A systematic evaluation of the relationship between vitamin D deficiency during pregnancy and PTB in 2016^[10]^ showed that pregnant women with vitamin D deficiency during pregnancy had an increased risk of PTB. According to the updated systematic evaluation on this issue in 2017,^[11]^ circulatory 25-OH D deficiency in pregnant women could increase the risk of PTB, and vitamin D supplementation alone during pregnancy can reduce the risk of PTB. Although systematic reviews have analyzed the relationship between vitamin D deficiency during pregnancy and PTB, no studies have shown a relationship between vitamin D deficiency during different pregnancies and PTB. Additionally, high-quality meta-analysis has been increasingly regarded as 1 of the key tools to obtain evidence.^[12,13]^ Therefore, we performed a meta-analysis combining all available data from available observational studies to obtain a more accurate estimate of the effect of vitamin D levels during different pregnancies on the risk of PTB.

## 2 Materials and methods

The Consortium on Vitamin D and Pregnancy is a collaboration of prospective birth cohorts that aims to study the association of the function of maternal vitamin D in different periods of gestation with adverse pregnancy and child outcomes. For the current study, we followed preferred reporting items for systematic reviews and meta-analyses (PRISMA)^[14–16]^ guidelines (see supplementary materials), which helped to improve the integrity of this review. A Measurement Tool to Assess Systematic Reviews (AMSTAR 2) was used to assess the methodological quality of this study.^[17,18]^ The study was approved by the Institutional Review Boards of Gansu Provincial Maternity and Child-Care Hospital.

### 2.1 Search strategies

To identify studies for inclusion, we conducted a systematic literature search for articles on the association of vitamin D with PTB published from the database's inception to February 7, 2020, without language restrictions, using the PubMed-Medline, EMBASE, the Cochrane Library, Web of Science, EBSCO, CBM, and CNKI databases. Additional relevant studies were identified from the list of references from the included publications. Search terms included a mix of medical subject headings (MeSH) and free-text words. We used the PICOS model,^[19]^ which means P (participant), I (intervention), C (comparison), O (outcome), S (study design); to determine the inclusion criteria, as follows: I (intervention)—“vitamin D, cholecalciferol, ergocalciferol, 25-hydroxy-vitamin D, 25 (OH)D,” O (outcome)—“premature birth, PTB, premature labor, preterm labor, premature delivery, preterm delivery, prematurity” (see supplementary materials).

### 2.2 Inclusion and exclusion criteria

Studies were selected only if they satisfied the following criteria: (1) they were cohort studies, case–control studies, and cross-sectional studies; (2) the population was pregnant women without chronic disease, HIV infection, or depressive symptoms; (3) they included pregnant women of any gestational age, and the duration of pregnancy was determined based on the date of the last menstruation, or by ultrasound; (4) maternal blood samples were taken for assays of 25 (OH)D in 3 periods: the first trimester, which extends through the completion of 14 weeks, the second, through 28 weeks, and the third, including the 29th through 42nd weeks of pregnancy^[20]^; (5) vitamin D deficiency was defined as a 25 (OH)D level below 20 ng/mL; (6) PTB was defined as delivery of a live born neonate before 37 weeks of gestation; (7) sufficient data were provided to calculate the effect of gestational 25 (OH)D status on PTB; and (8) studies published in English or Chinese. Exclusion criteria were as follows: (1) systematic reviews, meta-analyses, case report, letters, conference abstracts, etc; (2) animal experiments; (3) duplicate data; (4) vitamin D supplementation during pregnancy as a control study; and (5) the gestational week of blood sample was not clear, including not described or involving 2 period trimesters of pregnancy (ie, 12–24weeks).

### 2.3 Study selection and data extraction

All titles and abstracts from the search were cross-referenced to identify duplicates. Titles and abstracts were screened for a subsequent full-text review. After the full-text review, the papers included were retained for data extraction. From all the eligible studies, the following key information was extracted by means of a standard format: the first author's last name, year of publication, title and journal of publication, study design, the country (province or city), and time in which the study was performed, the source and number of participants, age of participants, gestational age at serum sampling, assay method of serum 25 (OH)D, diagnostic criteria of vitamin D deficiency, diagnosis criteria of PTB, and possible confounding factors in adjustment. Primary outcomes were the total level of vitamin D deficiency, total number of PTBs, and the level of vitamin D deficiency associated with PTBs.

During study selection and data extraction, 2 authors (RHL and PAQ) independently assessed the studies, and disagreements were resolved through discussions between them or with a third author (TY).

### 2.4 Quality assessment

A nine-star system based on the Newcastle–Ottawa Scale (NOS)^[21]^ was used to assess the quality of cohort studies and case–control studies in meta-analysis. The scoring system summarized 3 major aspects (selection, comparability, and outcome) and 8 detailed items. High-quality studies were defined as scoring 6 or more of 9 total points.^[22]^

The Agency for Healthcare Research and Quality (ARHQ) methodology checklist was used for cross-sectional studies, which included 11 items with a summary judgment.^[23,24]^

Two different authors (RHL and PAQ) independently appraised the risk of bias of the included studies, and disagreements were resolved by consensus or discussion with a third author (TY).

### 2.5 Statistical analysis

Binary data were combined and effect sizes were presented as ORs (odds ratios) with 95% CIs. Forest plots were generated to illustrate the study-specific effect sizes along with a 95% CI. Heterogeneity across studies was measured by the *Q*-test and the *I*^2^ statistic (degree of heterogeneity). If the *P*-value from the *Q*-test was less than 0.1 and/or the *I*^2^ was greater than 25%, heterogeneity across studies was presented. In detail, it was determined that the values of 25%, 50%, and 75% in the *I*^2^ test corresponded to low, moderate, and high levels of heterogeneity, respectively.^[25]^ We used a fixed-effect model (Mantel–Haenszel method) if there was no heterogeneity across studies; otherwise, the random effect model was applied.

Subgroup analysis was used to analyze the possible sources of heterogeneity. Sensitivity analysis was conducted by removing individual studies 1 by 1 to observe the influence of each study on the combined effect size. If 10 or more studies are included, Funnel plots and Begg tests^[26]^ were used to evaluate potential publication bias. All statistical analyses were performed using STATA/SE Version 12.0 (StataCorp, College Station, TX, USA). A *P*-value <.05 was considered as statistically significant for all tests except for the heterogeneity test, in which a *P*-value <.10 was used.

## 3 Results

### 3.1 Search results

**Figure 1:**
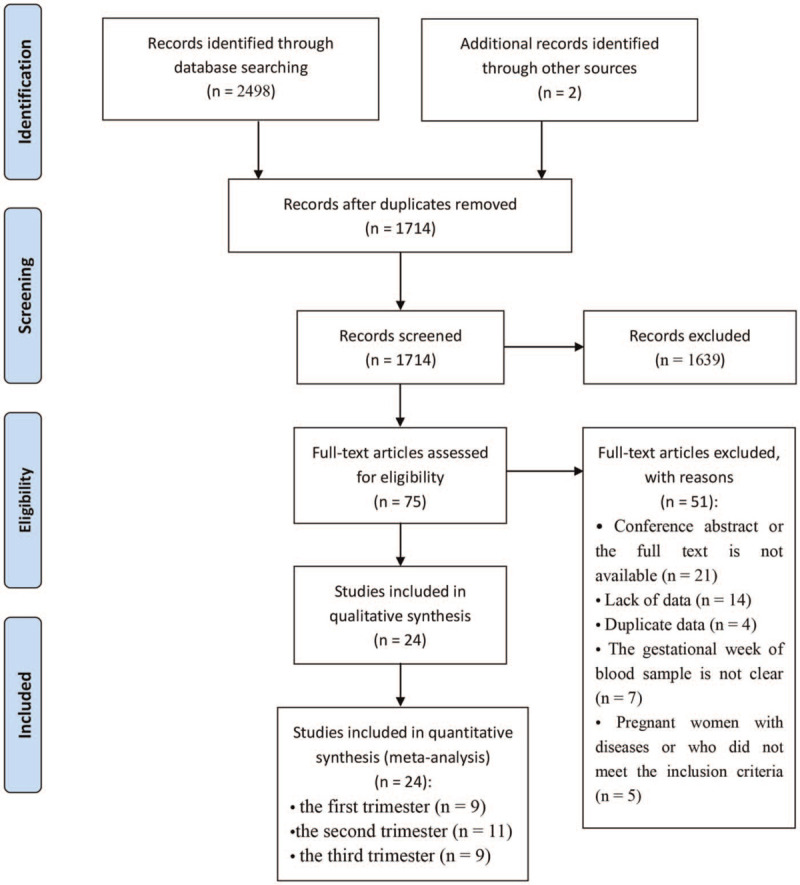
Preferred reporting items for systematic reviews and meta-analyses (PRISMA) flow diagram of study selection.

The detailed steps of the study selection are given as a PRISMA flow diagram in Figure 1. A total of 2498 abstracts were retrieved from the databases, and 2 studies were added from the references; 1639 were excluded after reading titles and/or abstracts, and 75 articles were subjected to a full-text review. After reading the full text, a total of 24 cohorts were invited to participate, which included 13 cohort studies,^[27–39]^ 7 case–control studies,^[40–46]^ and 4 cross-sectional studies.^[47–50]^ Because 3 studies^[29–31]^ of 24 measured serum 25 (OH)D concentrations at 2 or 3 periods of pregnancies, our meta-analysis included 9 studies^[28–31,36,39,41,44,46]^ in the first trimester, 11 studies^[27,30–35,37,38,40,43]^ in the second trimester, and 9 studies^[29–31,42,45,47–50]^ in the third trimester.

### 3.2 Study characteristics

The full list of studies included^[27–50]^ and their main characteristics are shown in Table 1. The included studies from the United States, Canada, Australia, New Zealand, Spain, Netherlands, Swedish, Poland, Brazil, Kenya, China, Singapore, and Thailand. A total of 9 studies^[28,30,31,33,37,40,48–50]^ were from Asian countries, 6^[36,41,43,45–47]^ from American countries, 5^[29,34,38,39,42]^ from European countries, 3^[27,35,44]^ from Oceanian countries, and 1 ^[32]^ was from African countries. Among 24 studies, 21^[27–46]^ cohort studies or case–control studies were appraised by NOS, resulting in scores above 6, while 4 ^[47–50]^ cross-sectional studies were appraised by the ARHQ methodology checklist, which also showed good quality (see supplementary materials).

**Table 1:** Characteristics of studies included in the meta-analysis. PTB: preterm birth.

First author's last name (year)	Country (province or city) of study	Study design	Time	Age	Gestational age at serum sampling	Assay method of serum 25 (OH)D	Diagnostic criteria of vitamin D deficiency	Diagnostic criteria of PTB	Adjustment
Ding (2018)^[28]^	China (Shanghai)	Cohort study	2015.1–2016.12	31 ± 3.5	≤90 d	CMIA	25 (OH)D <50 nmol/L	<37 wks	a, b, c
Bärebring (2018)^[29]^	Sweden (Gothenburg)	Cohort study	Fall, 2013 to spring, 2014	Not mentioned	8–12 wks, 32–35 wks	LC–MS/MS	25 (OH)D <30 nmol/L	<37 wks	c, e, f, g
Zhou (2017)^[30]^	China (Ma an-shan)	Cohort study	2013.5–2014.9	26.1 ± 3.7	<14 wks, 24–28 wks, >32 wks	RIA	25 (OH)D <20 ng/mL	28–36 + 6 wks	a, b, e, g, h, i, j, k
Chen (2017)^[31]^	China (Fujian)	Cohort study	2015.10.1–2016.9.30	29.09 ± 4.33	<13 wks, 24–28 wks, 32–34 wks	LC–MS/MS	25 (OH)D ≤20 ng/mL	<37 wks	a, l, m
Tabatabaei (2017)^[41]^	Canada (Quebec)	Case–control study	Not mentioned	aged ≥18 y	8–14 wks	LC–MS/MS	25 (OH)D <50 nmol/L	<37 wks	a, b, c, d, f, i, m
Flood-Nichols (2015)^[36]^	USA (Madigan)	Cohort study	2014	24.3 ± 4.4	5–12 wks	ELISA	25 (OH)D <50 nmol/L	<37 wks	b, c, d, f
Schneuer (2014)^[44]^	Australia (New South Wales)	Case–control study	2006.10–2007.9	33.1 ± 4.7	10–14 wks	AIA	25 (OH)D <50 nmol/L	<37 wks	a, b, c, e, f, i, m
Fernández-Alonso (2012)^[39]^	Spain (Almería)	Cohort study	2009.5.1–2010.4.30	Not mentioned	11–14 wks	ECLIA	25 (OH)D ≤20 ng/mL	<37 wks	b, c, d, f
Baker (2011)^[46]^	USA (North Carolina)	Case–control study	2004.11–2009.7	33.5 ± 1.75	11–14 wks	LC–MS/MS	25 (OH)D <50 nmol/L	23 0/7–34 6/7 wks	a, b, c, n
Qiu(2018) ^[40]^	China (Wenzhou)	Case–control study	2016.6–9	28.75 ± 4.25	About 20 wks	ECLIA	25 (OH)D < 50 nmol/L	<37 wks	a, b, m
Wilson (2018)^[27]^	Australian (Adelaide) and New Zealand (Auckland)	Cohort study	2004.11–2008.9	28 ± 6	15 ± 1 wks	CLIA	25 (OH)D <50 nmol/L	≤37 wks	a, b, c, d, f, i, j, p
Toko (2016)^[32]^	Kenya (Kisumu)	Cohort study	2011.6–2012.7	22.5 ± 6.6	19.9 ± 5.7 wks	ELISA	25 (OH)D <50 nmol/L	<37 wks	a, b
Ong (2016)^[33]^	Singapore	Cohort study	2009.6–2010.9	30.5 ± 5.1	26–28 wks	LC–MS/MS	25 (OH)D <50 nmol/L	<37 wks	a, b, d, f, h, k, q
Miliku (2016)^[34]^	Netherlands (Rotterdam)	Cohort study	2002.4–2006.1	29.7 ± 5.2	18.5–23.3 wks	LC–MS/MS	25 (OH)D <50 nmol/L	<37 wks	a, b, c, d, f, g, h, j, m, q, r, s
Boyle (2016)^[35]^	New Zealand (Auckland)	Cohort study	2005–2008	30.3 ± 4.7	15 wks	LC–MS/MS	25 (OH)D <50 nmol/L	<37 wks	b, d
Bodnar (2015)^[43]^	USA (Pittsburgh)	Case–control study	1999–2001, 2003, 2007–2010	Not mentioned	20 wks	LC–MS/MS	25 (OH)D <50 nmol/L	<37 wks	b, c, d, f, h, m, u
Zhou (2014)^[37]^	China (Guangzhou)	Cohort study	2010.9–2011.8	≥18 y	16–20 wks	ECLIA	25 (OH)D <20 ng/mL	<37 wks	a, b, o, z
Perez-Ferre (2012)^[38]^	Spain (Madrid)	Cohort study	2010.6.1–9.30	32.5 ± 1.75	24–28 wks	CLIA	25 (OH)D <20 ng/mL	<37 wks	a, b, d, f, y
Kassai (2018)^[47]^	Brazil (São Paulo)	Cross-sectional study	2016.3–2017.5	26.0 ± 7.3	At the time of the delivery admission	ECLIA	25 (OH)D <20 ng/mL	<37 wks	a, b, d, f, g, m, j, x
Bhupornvivat (2017)^[48]^	Thailand (Bangkok)	Cross-sectional study	2014.7.1–2015.5.31	28.9 ± 7.6	Prior to labor	ECLIA	25 (OH)D <20 ng/mL	<37 wks	a, b, d, l
Baczyńska-Strzecha (2017)^[42]^	Poland (Lodz)	Case–control study	2013–2015	30.5 ± 5.75	Prior to labor	ELISA	25 (OH)D <30 ng/mL	22–36.6 wks	a, b, v, w
Wang (2015)^[49]^	China (Kunming)	Cross-sectional study	2014.5–11	28.9 ± 7	Prior to labor	LC–MS/MS	25 (OH)D <20 ng/mL	<37 wks	d, g, h, t
Zhu (2015)^[50]^	China (Shenyang)	Cross-sectional study	2012.1.1–2013.1.1	31 ± 7	Prior to labor	ELISA	25 (OH)D <50 nmol/L	<37 wks	c
Dunlop (2012)^[45]^	USA (Nashville)	Case–control study	2003–2006	25.95 ± 6.0	At the time of the delivery admission	ELISA	25 (OH)D <20 ng/mL	22 0/7–36 6/7 wks	a, b, d, n, u

Most of the studies defined vitamin D deficiency as a serum 25 (OH)D below 50 nmol/L or 20 ng/mL, but 2 studies^[29,42]^ defined as 25 (OH)D below 30 ng/mL were also included because there were data about serum 25 (OH)D below 20 ng/mL; thus, the criteria for diagnosis and data extraction were agreed upon. Similarly, the majority of studies defined PTB as a gestational age <37 weeks and term birth as a gestational age ≥37 weeks, but 1 study^[46]^ defining gestational age <35 weeks were also included because data about gestational age <37 weeks were available.

These studies were carried on from 1999 to 2017, and published from 2012 to 2018. Of 24 studies, 7 different assay methods were used to measure maternal vitamin D levels, which is in accordance with the Vitamin D standardization program (VDSP).^[51]^ Importantly, liquid chromatography–tandem mass spectrometry (LC–MS/MS) is considered to be the gold standard for the determination of vitamin D.^[51,52]^

All of these studies described the association between vitamin D deficiency during pregnancy and PTB, whether negative or positive.

### 3.3 Meta-analysis

**Figure 2:**
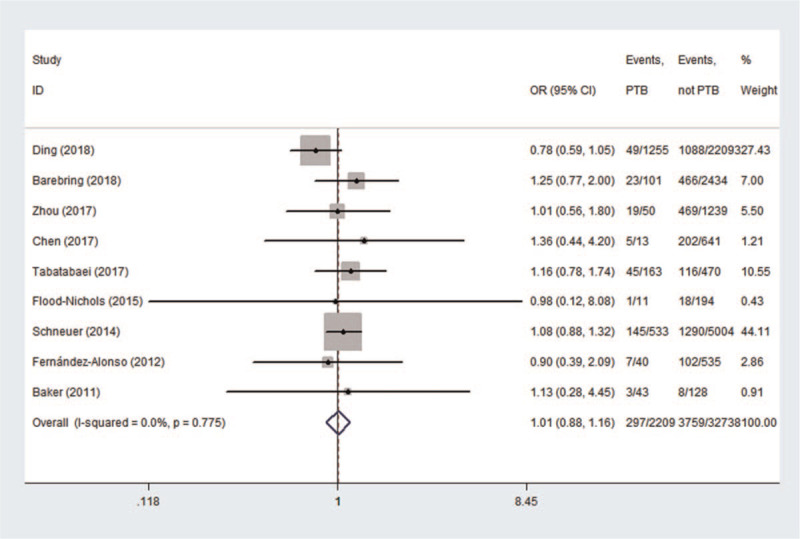
The meta-analysis of the association between maternal vitamin D deficiency in the first trimester and PTB. PTB = preterm birth.

**Figure 3:**
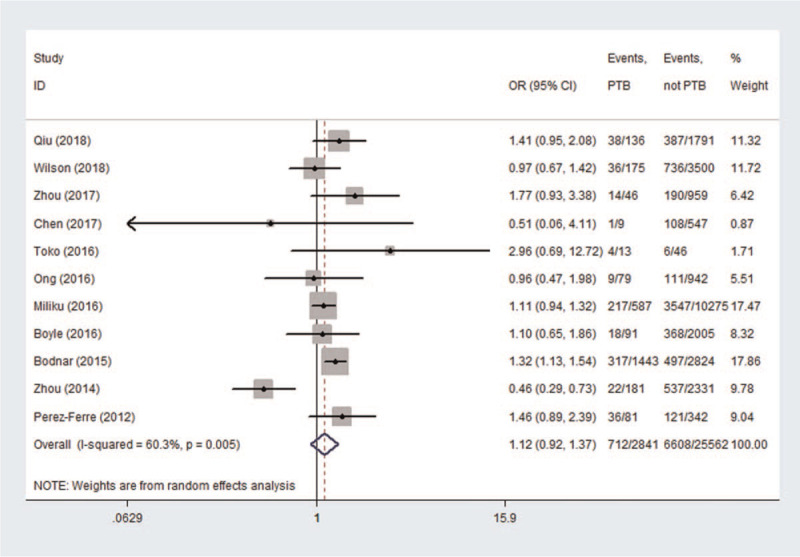
The meta-analysis of the association between maternal vitamin D deficiency in the second trimester and PTB. PTB = preterm birth.

**Figure 4:**
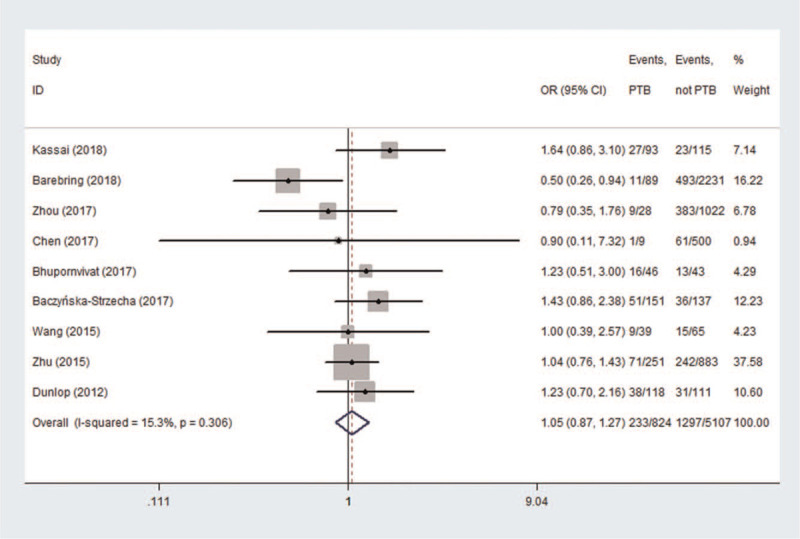
The meta-analysis of the association between maternal vitamin D deficiency in the third trimester and PTB. PTB = preterm birth.

According to the lower serum 25 (OH)D concentration (<50 nmol/L or <20 ng/mL) which diagnosed vitamin D deficiency, the results of the meta-analysis appear to be inconsistent in the different periods of pregnancy. In Figure 2, the association between maternal vitamin D deficiency in the first trimester and PTB was not statistically significant (OR = 1.01, 95%CI: 0.88, 1.16, *P* = .876). In Figure 3, the pregnant women with vitamin D deficiency in the second trimester showed no statistical significance regarding the risk of developing PTB (OR = 1.12, 95%CI (0.92, 1.37), *P* = .249) in a random effect model. In Figure 4, the association between maternal vitamin D deficiency in the third trimester and PTB was not statistically significant (OR = 1.05, 95%CI: 0.87, 1.27, *P* = .602).

### 3.4 Sensitivity and subgroup analysis

**Figure 5:**
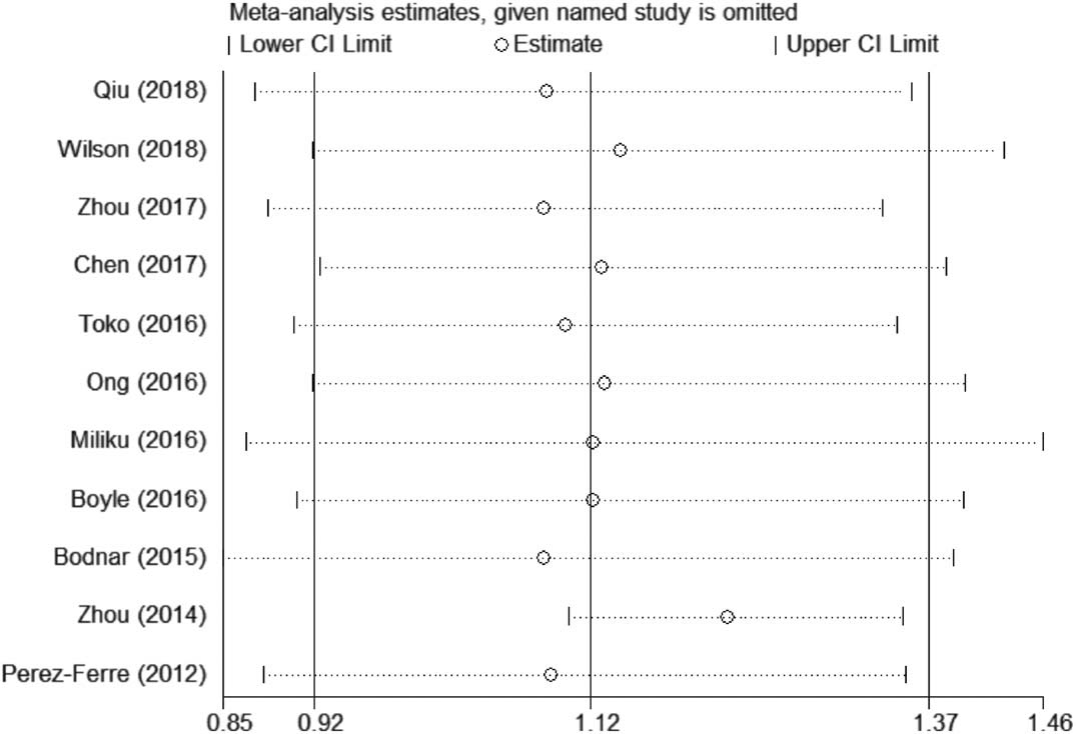
Sensitivity analyses of the association between maternal vitamin D deficiency in the second trimester and PTB. PTB = preterm birth.

**Figure 6:**
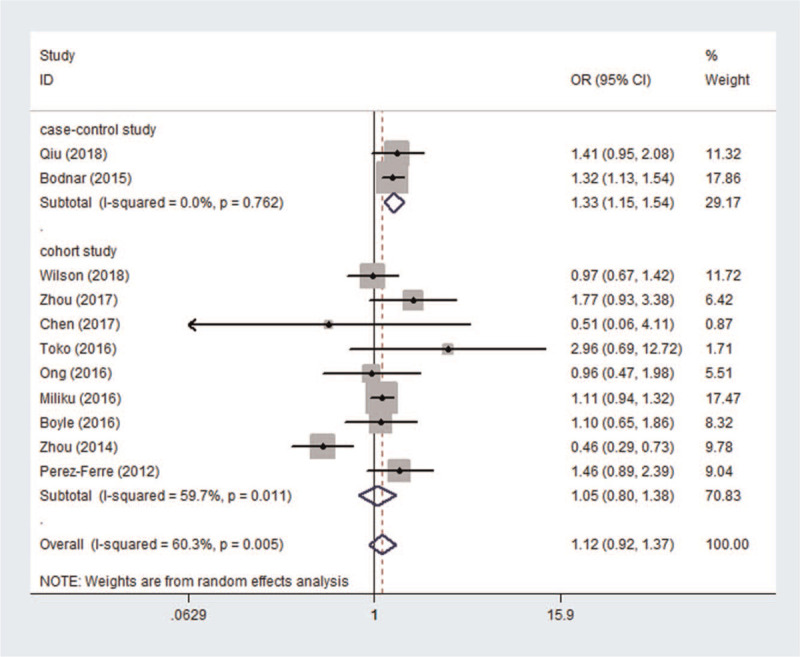
The results of subgroup analysis according to different study designs.

**Figure 7:**
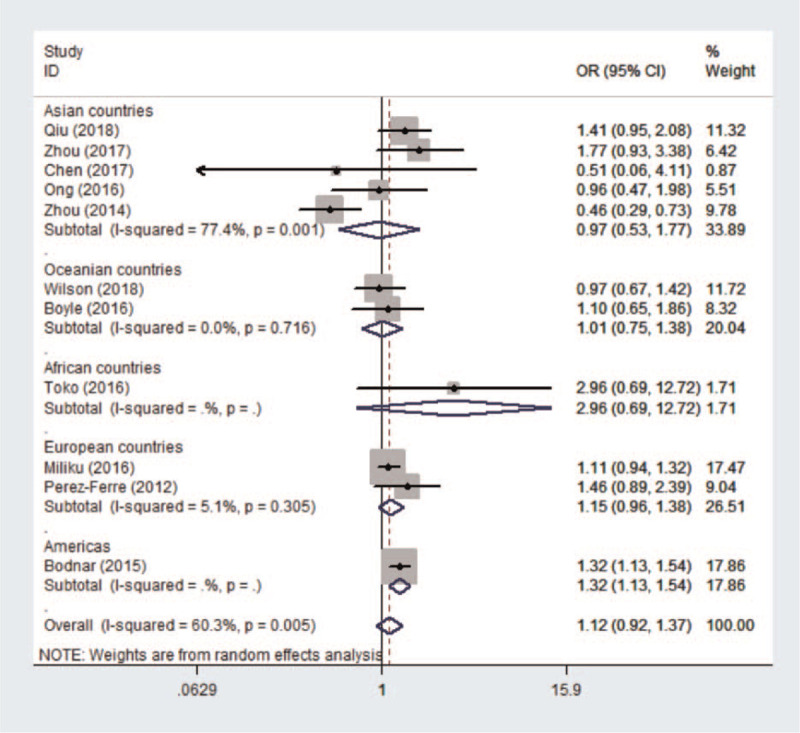
The results of subgroup analysis according to countries from different continents.

In the meta-analysis of the association between maternal vitamin D deficiency in the first and third trimesters and PTB, tests revealed no heterogeneity (*I*^2^ = 0, *P* > .1; *I*^2^ = 15.3%, *P* > .1); thus, a fixed-effect model was used for meta-analysis. In the meta-analysis of the relationship between maternal vitamin D deficiency in the second trimester and PTB, heterogeneity tests revealed that *I*^2^ = 60.3% (*P* < .1), indicative of moderate heterogeneity; thus, a random effect model was used. The sensitivity analyses, shown in Figure 5, indicated significant changes in the result when the study by Zhou et al^[37]^ was excluded. Subgroup analyses were performed according to the study design and the continents with relevant countries. A significant association was identified in 2 case–control studies^[40,43]^ between maternal vitamin D deficiency and PTB in Figure 6 (OR = 1.33, 95%CI: 1.15, 1.54, *P* = 0.000). Stratifying the countries from different continents in Figure 7, 5 studies conducted in Asian countries^[30,31,33,37,40]^ showed high heterogeneity (*I*^2^ = 77.4% (*P* < .1)), while the study by Bodnar et al in the Americas^[43]^ revealed a statistically significant protective effect among pregnant women, with an OR of 1.32 (95% CI 1.13–1.54, *P* = .001). A random-effect model was used for this meta-analysis because of the heterogeneity in all subgroup analyses.

### 3.5 Publication bias

The Begg funnel plot of the effect of vitamin D deficiency in the second trimester on PTB appeared to be symmetrical, as shown in Figure 8. No significant publication bias was detected (*P* = .69). Regarding other outcomes of vitamin D deficiency in the first or third trimester and PTB, due to the limited number of studies, publication biases cannot be excluded.

## 4 Discussion

In this review, 13 of the 24 observational studies clearly reported no effect of vitamin D deficiency during pregnancy on PTB, no matter whether in early pregnancy, in middle pregnancy, or in late pregnancy.^[27,28,31–33,35,36,39,44–46,48,49]^ However, other studies showed different results. Five of all studies included showed that lower 25 (OH)D levels in middle pregnancy are associated with PTB, and even that there is a protective association between maternal vitamin D sufficiency and PTB.^[30,34,38,40,43]^ Kassai et al study show that mothers who delivered preterm babies had lower 25 (OH)D concentrations prior to delivery compared to women who had given birth at the full-term of their pregnancy.^[47]^ Baczyńska-Strzecha et al data confirmed that severe vitamin D deficiency (<10 ng/mL) in late pregnancy may be a factor increasing the risk of PTB.^[42]^ More specially, Zhou et al study showed that PTB with a high level of vitamin D in the second trimester of pregnancy had a higher prevalence than that in low and medium-level groups, possibly related to the older age and higher BMI of the high-level group.^[37]^

There was no observed heterogeneity in studies of vitamin D deficiency and PTB in early and late pregnancy, and the results showed there is little association between them. However, moderate heterogeneity existed in the study of the second period of pregnancy, which revealed a positive correlation between vitamin D deficiency and PTB. The subgroup of analysis by study design showed 2 cohort studies^[27,34]^ had a non-ignorable effect. Wilson et al study compared and combined 2 distinct populations of pregnant women living at similar latitudes, and found that circulating 25 (OH)D was different between women recruited in Adelaide compared to women recruited in Auckland.^[27]^ A large-scale prospective cohort including 7098 mothers and their offspring showed lower maternal 25 (OH)D concentrations during mid-pregnancy, which were associated with a higher risk of PTB, which is different from the majority of studies; this may be due to the universality of the study population and the larger sample size, which is likely to be the cause of heterogeneity.^[34]^ Qiu et al study is the main source of heterogeneity^[40]^ in the subgroup of analysis by continents and countries, but there was no heterogeneity shown in the subgroup of analysis by study design. The results of the meta-analysis did not change after removing the papers that caused heterogeneity.

In our meta-analysis, diagnostic criteria of vitamin D deficiency and PTB need to be unified first. Vitamin D deficiency is very common all over the world, but it varies by different cutoffs. The American Institute of Medicine considers a serum level of 25 (OH)D above 20 ng/mL (50 nmol/L) to be sufficient for pregnant women,^[53]^ whereas the American Endocrine Society recommends a serum level of 25 (OH)D above 30 ng/mL (75 nmol/L) to be sufficient.^[54]^ Most studies were classified according to the same criteria: vitamin D deficiency (<20 ng/mL), insufficient (20–30 ng/mL), and sufficient (≥30 ng/mL). Bärebring et al study^[29]^ and Baczyńska-Strzecha et al study^[42]^ categorized vitamin D deficiency as below 30 ng/mL; the cause for this is perhaps that some European countries were accustomed to using the endocrine society criteria. Luckily, data below 20 ng/mL were also available, so they were included. Similarly, Baker et al study^[39]^ defined PTB as less than 35 weeks, and we could extract data about less than 37 weeks, as with all of the other studies.

Vitamin D levels are usually assessed by measuring the compound 25-hydroxyvitamin D (25 (OH)D), which is the circulating form of vitamin D.^[55]^ According to our statistics, 7 assay methods of serum 25 (OH)D were used in the 24 studies, including chemiluminescence microparticle immune assay, LC–MS/MS, radioimmunoassay, enzyme-linked immunosorbent assay, automated immunoassay, electrochemiluminescence immunoassay, chemiluminescence immunoassay. As a fact, there is substantial within-assay variation in 25 (OH)D measurement and even greater between-assay variability. The mean inter-assay and intra-assay coefficient of variations for serum 25 (OH)D concentration in our 24 studies was about 5%. Such assay variation clearly confounds attempts to define what constitutes the diagnosis of hypovitaminosis D. Therefore, to develop and implement evidence-based clinical guidelines, 25 (OH)D measurement must be standardized in both clinical and research laboratories; thus, the VDSP was born.^[51]^ The VDSP does not mandate or suggest a single analytic approach but requires researchers to abide by the standardization steps. The above detection methods adopted in our studies basically meet these requirements, and so although the measurement methods are different, the heterogeneity is lower, which provides support for the extrapolation of meta-analysis results.

As is well known, vitamin D has an important role in maintaining an adequate level of minerals through its influence on calcium and phosphate metabolism for bone mineralization and metabolic functions. The association between vitamin D deficiency and bone diseases such as rickets and osteoporosis is well recognized; however, increasingly, a relationship between vitamin D deficiency and other conditions have been identified.^[55]^ In recent years, studies on vitamin D levels in pregnancy and pregnancy outcomes have become increasingly extensive. The compound 1,25 (OH)_2_D as the active form of vitamin D has non-genomic and genomic effects through its action on vitamin D receptors.^[56]^ The nongenomic effects of vitamin D occur rapidly; examples include protein kinase activation and the activation of ion channels.^[57]^ The genomic effects occur over a long period of time and are mediated by 1,25 (OH)_2_D via the nuclear VDR to initiate and regulate gene expression, which is the engine driving fetal development.^[58]^ Consequently, vitamin D deficiency is associated with increased rates of fetal miscarriage, preeclampsia, gestational diabetes, and impaired fetal and childhood growth and development.^[56]^ On the other hand, vitamin D can also affect the pathophysiology of PTB by affecting inflammatory and immunomodulatory processes.^[59]^ It is responsible for initiating the adequate function of toll-like receptors in innate immune responses. Patients with vitamin D deficiency are more susceptible to infection due to the impaired induction of the toll-like antimicrobial peptide cathelicidin in macrophages.^[60]^

As mentioned earlier, several observational studies have drawn different or even opposite conclusions regarding the relationship between vitamin D deficiency and PTB. This may be due to differences in the study population, region, ethnicity, etc, and so the results should be interpreted with caution. Some studies excluded from this meta-analysis due to the inclusion and exclusion criteria also illustrate certain conclusions. A large prospective population-based birth cohort study set up in several geographical areas of Spain^[61]^ did not find any evidence of an association of maternal circulating 25 (OH)D3 concentration in pregnancy with PTB, although there was possible selection bias because they did not measure circulating 25 (OH)D2 concentrations, but only the D3 form, which normally makes up the majority (90%) of 25 (OH)D. Choi et al data^[62]^ indicated a high prevalence of vitamin D deficiency among pregnant women in Korea, but no significant associations between vitamin D deficiency and PTB were observed in Korean pregnant women. Unfortunately, this study did not specify when the blood was collected to assay 25 (OH)D. On the contrary, the study by Shibata et al^[63]^ suggested a high prevalence of vitamin D deficiency in perinatal pregnant Japanese women throughout the year, which seems to affect bone metabolism and to be associated with threatened PTB. Thota et al data^[64]^ from Tennessee, USA showed that in addition to having low levels of serum 25 (OH)D, African American women also have lower levels of 1,25-(OH)_2_D (the active form of vitamin D) compared to Caucasian women, and they further revealed that the levels of 1,25-(OH)_2_D at the time of delivery were significantly lower in women who delivered at preterm compared to their respective term counterparts, for both races. Hence, these results indicated that vitamin D deficiency is a risk factor for PTB in African American and Caucasian women. A meta-analysis of 11 observational studies proved an association between maternal vitamin D levels and PTB.^[10]^ In the observational studies, vitamin D concentration was measured in different stages of pregnancy, although not all studies were adjusted for confounders and the definition of PTB was not consistent between the studies.

The advantages of our meta-analysis are as follows: firstly, all studies included are middle to high quality, and they were adjusted for their most important factor or any additional factor confounders; secondly, we divided them into the first trimester, the second trimester, and the third trimester of pregnancy by bleeding time, which is also our innovation; thirdly, we unified the diagnostic criteria for vitamin D deficiency and PTB; and finally, in addition to the published literature, we also included some gray papers, such as Master's theses.

The limitation of our study is that although vitamin D supplementation as a case–control was explicitly excluded by our inclusion and exclusion criteria, it was not possible to accurately assess vitamin D intake or whether women in a natural pregnancy took vitamin D supplements on their own during pregnancy. Certainly, vitamin D deficiency in the second trimester was most associated with PTB, regardless of whether vitamin D was added in the first trimester.

## 5 Conclusions

Previous studies have concluded that vitamin D deficiency is common worldwide and is associated with many pregnancy outcomes.^[65]^ The relationship between vitamin D deficiency and PTB has been widely investigated in recent years. The evidence presented in our meta-analysis suggested that vitamin D deficiency in the second trimester of pregnancy is likely associated with an increased risk of PTB, and that there is little correlation between vitamin D deficiency in the first trimester and the third trimester of pregnancy and PTB. However, further research should be conducted on vitamin D intake during pregnancy to better determine the risks and benefits associated with such interventions and the potential public health implications.

## Author contributions

RHL, TY, and KHY designed the protocol of the systematic review. RHL and PAQ did the literature search, selected studies for inclusion, extracted data for analysis, and performed quality checks. RHL and WWQ performed the statistical analysis and interpreted the data. PJY, KHY, and BY checked the statistical methodology. RHL wrote the first draft, and all authors revised the manuscript for important intellectual content. All authors approved the final draft.

**Data curation:** Wen-Wen Qiu.

**Methodology:** Tao Yuan, Pei-Jing Yan, Ying Wei, Bin Yi.

**Writing – original draft:** Rui-Han Lian, Ping-An Qi.

**Writing – review & editing:** Ya-Guang Hu, Ke-Hu Yang.

## Supplementary Material

Supplemental Digital Content

## Supplementary Material

Supplemental Digital Content
