# Supplementary material for: Systematic review and meta-analysis of vitamin D deficiency in different pregnancy on preterm birth: Deficiency in middle pregnancy might be at risk
Source: Medicine (Baltimore). 2021 Jun 18;100(24):e26303. doi: 10.1097/MD.0000000000026303 (PMC8213249; doi:10.1097/MD.0000000000026303)
Supplement: Supplemental Digital Content [file medi-100-e26303-s002.doc]

**S2. For example: Search strategy used for PubMed-Medline database.**

| #1 | Search (("Vitamin D"[Mesh]) OR "Cholecalciferol"[Mesh]) OR "Ergocalciferols"[Mesh] | 56197 |
| --- | --- | --- |
| #2 | Search ((((Vitamin D[Title/Abstract]) OR Cholecalciferol[Title/Abstract]) OR Ergocalciferol[Title/Abstract]) OR 25-hydroxy-vitamin D[Title/Abstract]) OR 25(OH)D[Title/Abstract] | 19230 |
| #3 | Search #1 OR #2 | 61730 |
| #4 | Search (("Premature Birth"[Mesh]) OR "Obstetric Labor, Premature"[Mesh]) OR "Infant, Premature"[Mesh] | 74593 |
| #5 | Search ((((((Premature Birth[Title/Abstract]) OR Preterm Birth[Title/Abstract]) OR Premature Labor[Title/Abstract]) OR Preterm Labor[Title/Abstract]) OR Premature Delivery[Title/Abstract]) OR Preterm Delivery[Title/Abstract]) OR Prematurity[Title/Abstract] | 47141 |
| #6 | Search #4 OR #5 | 99050 |
| #7 | Search #3 AND #6 | 371 |

**S3. Quality assessment of case-control studies by the Newcastle-Ottawa Scale (total 9 points).**

| study | Selection | | | |  | Comparability | |  | Exposure | | | total score |
| --- | --- | --- | --- | --- | --- | --- | --- | --- | --- | --- | --- | --- |
|  | ① | ② | ③ | ④ |  | ⑤A | ⑤B |  | ⑥ | ⑦ | ⑧ |  |
| Tabatabaei(2017) | ★ | ★ | － | ★ |  | ★ | ★ |  | ★ | ★ | － | 7★ |
| Schneuer(2014) | ★ | ★ | － | ★ |  | ★ | ★ |  | ★ | ★ | － | 7★ |
| Baker(2011) | ★ | ★ | － | ★ |  | ★ | ★ |  | ★ | ★ | － | 7★ |
| Qiu (2018) | ★ | ★ | － | ★ |  | ★ | － |  | ★ | ★ | － | 6★ |
| Bodnar (2015) | ★ | ★ | － | ★ |  | ★ | ★ |  | ★ | ★ | ★ | 8★ |
| Baczyńska-Strzecha (2017) | ★ | ★ | － | ★ |  | ★ | ★ |  | ★ | ★ | － | 7★ |
| Dunlop (2012) | ★ | ★ | － | ★ |  | ★ | ★ |  | ★ | ★ | － | 7★ |
| ① is the case definition adequate ② representativeness of the cases ③ selection of controls ④ definition of controls ⑤A study controls for the most important factor ⑤B study controls for any additional factor ⑥ ascertainment of exposure ⑦ same method of ascertainment for cases and controls ⑧ non-response rate | | | | | | | | | | | | |
|  |  |  |  |  |  |  |  |  |  |  |  |  |

**S4. Quality assessment of cohort studies by the Newcastle-Ottawa Scale (total 9 points).**

| study | Selection | | | |  | Comparability | |  | Exposure | | | total score |
| --- | --- | --- | --- | --- | --- | --- | --- | --- | --- | --- | --- | --- |
|  | ① | ② | ③ | ④ |  | ⑤A | ⑤B |  | ⑥ | ⑦ | ⑧ |  |
| Ding(2018) | ★ | ★ | ★ | ★ |  | ★ | － |  | ★ | ★ | ★ | 8★ |
| Bärebring(2018) | ★ | ★ | ★ | ★ |  | － | ★ |  | ★ | ★ | ★ | 8★ |
| Zhou(2017) | ★ | ★ | ★ | ★ |  | ★ | － |  | ★ | ★ | ★ | 8★ |
| Chen(2017) | ★ | ★ | ★ | ★ |  | － | ★ |  | ★ | ★ | ★ | 8★ |
| Flood-Nichols(2015) | ★ | ★ | ★ | ★ |  | － | ★ |  | ★ | ★ | ★ | 8★ |
| Fernández-Alonso(2012) | ★ | ★ | ★ | ★ |  | － | ★ |  | ★ | ★ | ★ | 8★ |
| Wilson (2018) | ★ | ★ | ★ | ★ |  | ★ | ★ |  | ★ | ★ | ★ | 9★ |
| Toko (2016) | ★ | ★ | ★ | ★ |  | ★ | － |  | ★ | ★ | ★ | 8★ |
| Ong (2016) | ★ | ★ | ★ | ★ |  | ★ | ★ |  | ★ | ★ | ★ | 9★ |
| Miliku (2016) | ★ | ★ | ★ | ★ |  | ★ | ★ |  | ★ | ★ | ★ | 9★ |
| Boyle (2016) | ★ | ★ | ★ | ★ |  | － | ★ |  | ★ | ★ | ★ | 8★ |
| Zhou (2014) | ★ | ★ | ★ | ★ |  | ★ | ★ |  | ★ | ★ | ★ | 9★ |
| Perez-Ferre (2012) | ★ | ★ | ★ | ★ |  | ★ | － |  | ★ | ★ | ★ | 8★ |
| ① representativeness of the exposed cohort ② selection of the non exposed cohort ③ ascertainment of exposure ④ demonstration that outcome of interest was not present at start of study ⑤A study controls for the most important factor ⑤B study controls for any additional factor ⑥ assessment of outcome ⑦ was follow-up long enough for outcomes to occur ⑧ adequacy of follow up of cohorts | | | | | | | | | | | | |
|  |  |  |  |  |  |  |  |  |  |  |  |  |
|  |  |  |  |  |  |  |  |  |  |  |  |  |

**S5. Quality assessment of cross-sectional studies by the Agency for Healthcare Research and Quality methodology checklist.**

| study | ⑴ | ⑵ | ⑶ | ⑷ | ⑸ | ⑹ | ⑺ | ⑻ | ⑼ | ⑽ | ⑾ |
| --- | --- | --- | --- | --- | --- | --- | --- | --- | --- | --- | --- |
| Kassai (2018) | Yes | Yes | Yes | not clear | No | Yes | Yes | Yes | not clear | Yes | not clear |
| Bhupornvivat (2017) | Yes | Yes | Yes | not clear | No | Yes | not clear | Yes | not clear | Yes | not clear |
| Wang (2015) | Yes | Yes | Yes | not clear | No | Yes | Yes | Yes | Yes | Yes | not clear |
| Zhu (2015) | Yes | Yes | Yes | not clear | No | Yes | Yes | Yes | Yes | Yes | not clear |
| ⑴ define the source of information (survey, record review) ⑵ list inclusion and exclusion criteria for exposed and unexposed subjects (cases and controls) or refer to previous publications ⑶ indicate time period used for identifying patients ⑷ indicate whether or not subjects were consecutive if not population-based ⑸ indicate if evaluators of subjective components of study were masked to other aspects of the status of the participants ⑹ describe any assessments undertaken for quality assurance purposes (e.g., test/retest of primary outcome measurements) ⑺ explain any patient exclusions from analysis ⑻ describe how confounding was assessed and/or controlled ⑼ if applicable, explain how missing data were handled in the analysis ⑽ summarize patient response rates and completeness of data collection ⑾ clarify what follow-up, if any, was expected and the percentage of patients for which incomplete data or follow-up was obtained | | | | | | | | | | | |
|
|
|
|
|
|
|
